# Endometriosis Presenting as Hydronephrosis

**DOI:** 10.1100/tsw.2005.103

**Published:** 2005-10-16

**Authors:** Ahmed Bakheet Zaharani, G.V. Soundra Pandyan

**Affiliations:** Department of Urology, Assir Central Hospital, King Khalid College of Medical Sciences, Abha, Saudi Arabia

**Keywords:** endometriosis, ureteric obstruction, hormonal treatment, uretero-ureterostomy

## Abstract

The most serious urological complication of endometriosis is hydronephrotic renal atrophy secondary to ureteric involvement. As only half of these patients are symptomatic, it is commonly diagnosed late and more by the clinicians awareness and suspicion of this entity. We report a case of an unmarried young female who presented primarily with left loin pain of 2-year duration. She was found to have lower ureteric stricture by an IVU done by her referring doctor. Further workup at our center showed that she had pelvic endometriosis with hydronephrosis secondary to extrinsic ureteric endometriosis. She had a first-degree relative with the same disease. She had no menstrual problems. Diagnostic laparoscopy, biopsy of the lesion, ureteric dilatation with stenting, along with hormonal treatment was given to her as first line of treatment. There was no improvement of the ureteric obstruction even after 6 months of treatment. Finally, surgical excision of the endometrioma, left oophorectomy, along with resection of the ureteric stricture with uretero-ureterostomy was done. This case report includes details of her further management and outcome along with a brief review of literature.

## CASE REPORT

An unmarried, 27-year-old female was referred to the urology clinic as a case of left lower ureteric stricture, diagnosed by an IVU (intravenous urogram) at a peripheral hospital. She had recurrent attacks of colicky, left loin pain for 2 years. As it was getting worse for the last 2 months, she sought a medical opinion. There was no history of hematuria, dysuria, or frequency of urine. She also complained of dull pain in the suprapubic area. Her menstrual cycle was normal with no relation of the pain to it. She had no other medical illness. Her elder sister was a known case of pelvic endometriosis. Physical examination was normal. There was mild tenderness in the left loin, but no palpable lumps. Rectal and vaginal examinations were deferred as she was unmarried. Routine blood tests and renal functions were normal. Urine analysis showed five to six red blood cells and seven to ten pus cells per high power field. Urine culture was normal. Ultrasound examination (USG) of the abdomen and pelvis revealed left hydroureteronephrosis with no evidence of calculi. Cystic lesions were seen in both the ovaries and in the rectovesical pouch. IVU showed a left lower ureteric stricture about 1 cm long with proximal dilatation (Fig. 1). Computed tomography (CT) scan showed multiple septations in these ovarian cysts (Fig. 2).

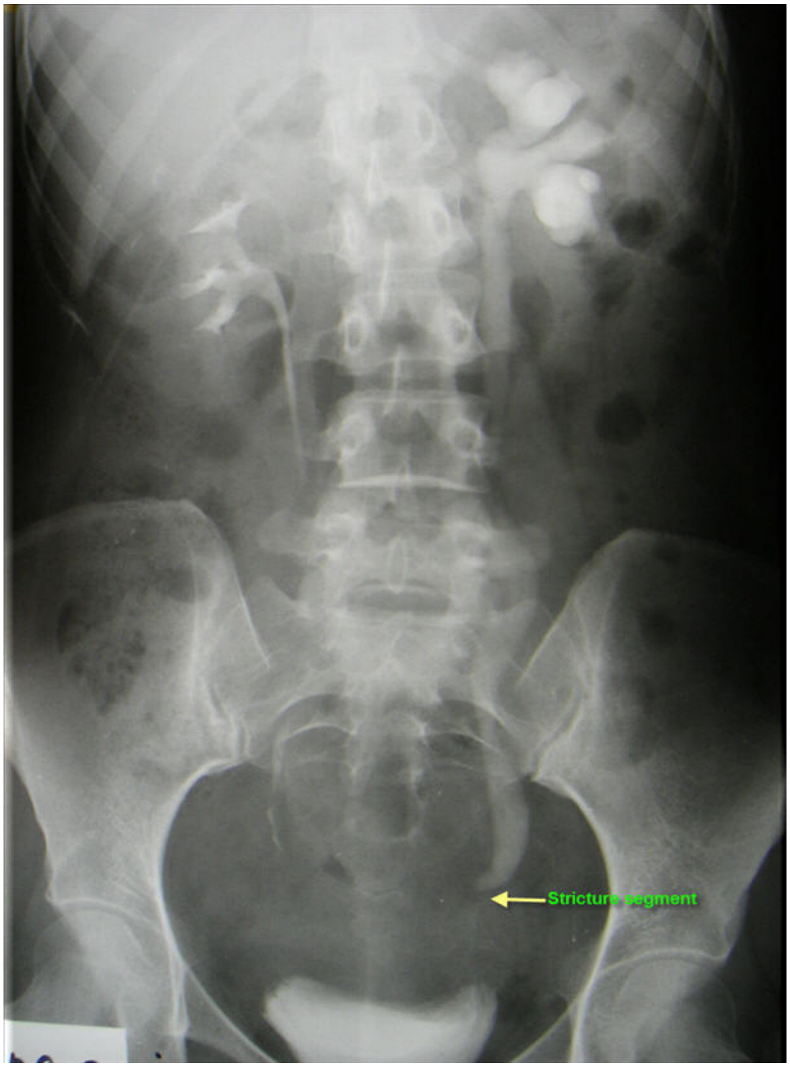

**FIGURE 1.** IVU showing left lower ureteric stricture with proximal dilatation.

Periureteric encasement of the left lower ureter by the lesion with proximal dilatation was also noted. Next we did a Cancer Antigen–125 (CA-125) assay, which was found to be elevated (86 U/ml). On cystoscopy, the urethra and bladder were normal. Left retrograde pyelogram (RGP) showed a stricture in the lower ureter just above its insertion into the bladder. A double J stent (JJ), 8/28 F, was inserted, following which gynecological opinion was sought. Diagnostic laparoscopy done by the gynecologist revealed Stage-III pelvic endometriosis. Biopsy from the lesions was taken and the histopathology confirmed endometriosis. Considering the age and marital status of the patient, conservative treatment was opted as a primary resort. Leuprolide acetate, 3.5 mg intramuscular, was started on a monthly basis with regular follow-up of the patient. Periodic cystoscopy, RGP, and change of JJ stent were done at three monthly intervals. She developed amenorrhea following this treatment. The patient's loin pain reduced, but RGP showed worsening of the ureteric stricture. During her second follow-up 6 months later, ureteric dilatation up to 10F and JJ reinsertion was done. At the 9-month follow-up, the patient was much concerned about her amenorrhea; RGP repeated showed further narrowing of the stricture. Ureteroscopy done at this juncture showed no evidence of intrinsic ureteral endometriosis (UE), but the stricture was more rigid and narrower. The decision for exploratory laparotomy was taken.

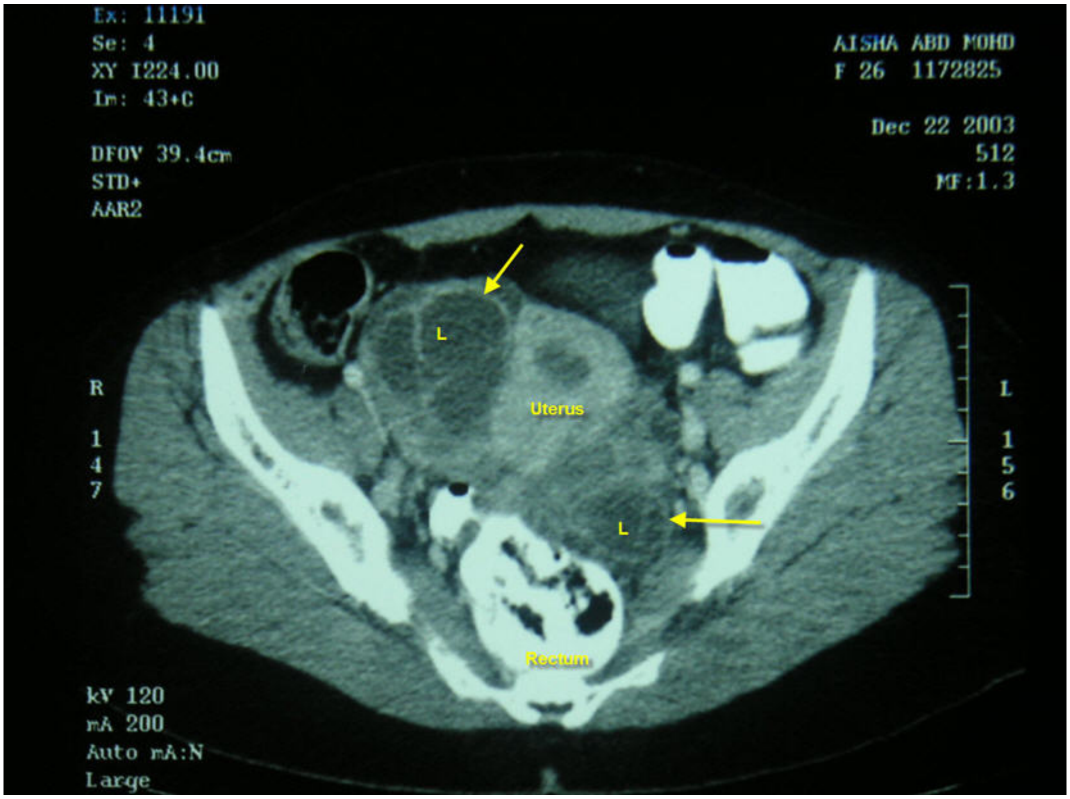

**FIGURE 2.** CT scan showing multiple septated cystic lesions in both the ovaries.

We followed up with magnetic resonance imaging (MRI), which showed bilateral cystic lesions in both ovaries and in the rectovesical pouch with left lower periureteric fibrosis associated with proximal dilatation (Fig. 3). Laparotomy revealed pelvic endometriosis involving both ovaries' left cardinal ligament, with dense fibrosis and narrowing of lower ureter proximal to its bladder insertion. The endometriomas appeared like yellowish-brown, tense, cystic lesions (Fig. 4). Excision of the right ovarian cystic part of the lesion, left oophorectomy, along with excision of the endometrioma in the left cardinal ligament was done. Chocolate-brown, thick fluid was seen in these cystic lesions while excising (Fig. 5). Exploration of the left lower ureter showed dense periureteric fibrosis with 1-cm-long narrowed segment. Excision of this narrowed segment and uretero-ureterostomy was carried out. Histopathology confirmed endometriosis in the cystic lesions excised (Fig. 6), but the ureteric segment showed only features of chronic inflammation and fibrosis with areas of squamous metaplasia. Patient did well postoperatively and has been followed up regularly for the last 9 months. IVU and renal scan done 3 months postoperatively showed no more evidence of ureteric obstruction. CA-125 assay came down to 41 U/ml. Her cycles have been regular and she has being doing well to date.

## DISCUSSION

Endometriosis is defined as the presence of ectopic endometrial glands outside the normal confines of the uterine cavity. Endometriosis was first described by Russel in 1955. Ureteric involvement by endometriosis was first described by Cullen in 1917. The overall incidence of urinary tract involvement is found to be 1% and ureteric involvement as low as 0.1–0.4%[1]. Urologists see patients with endometriosis who present with only pelvic pain and bladder complaints or the others who present with the complications of endometriosis such as ureteral obstruction or hematuria. The diagnosis of vesical or

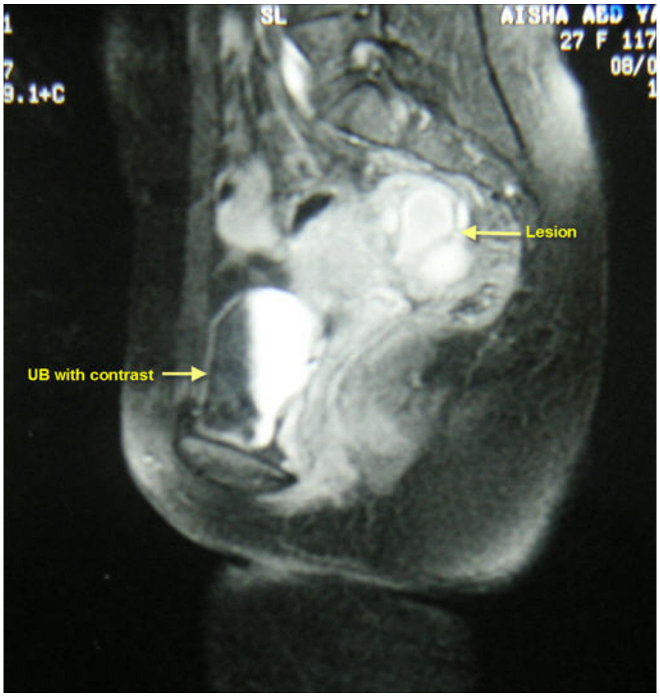

**FIGURE 3.** MRI showing cystic lesion in ovary.

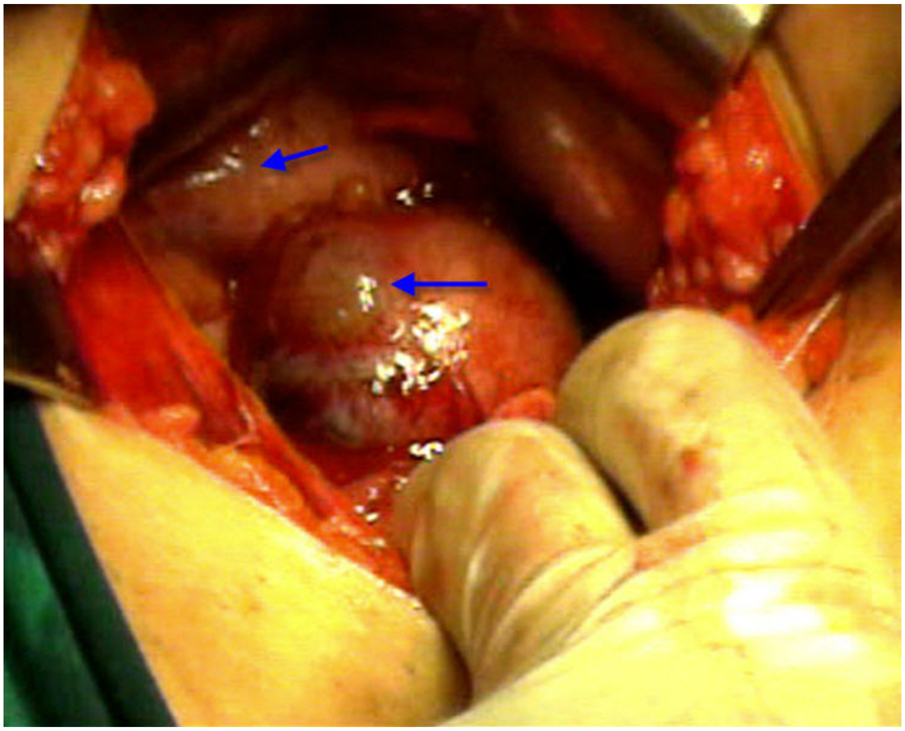

**FIGURE 4.** Yellowish-brown, tense, cystic endometriomas.

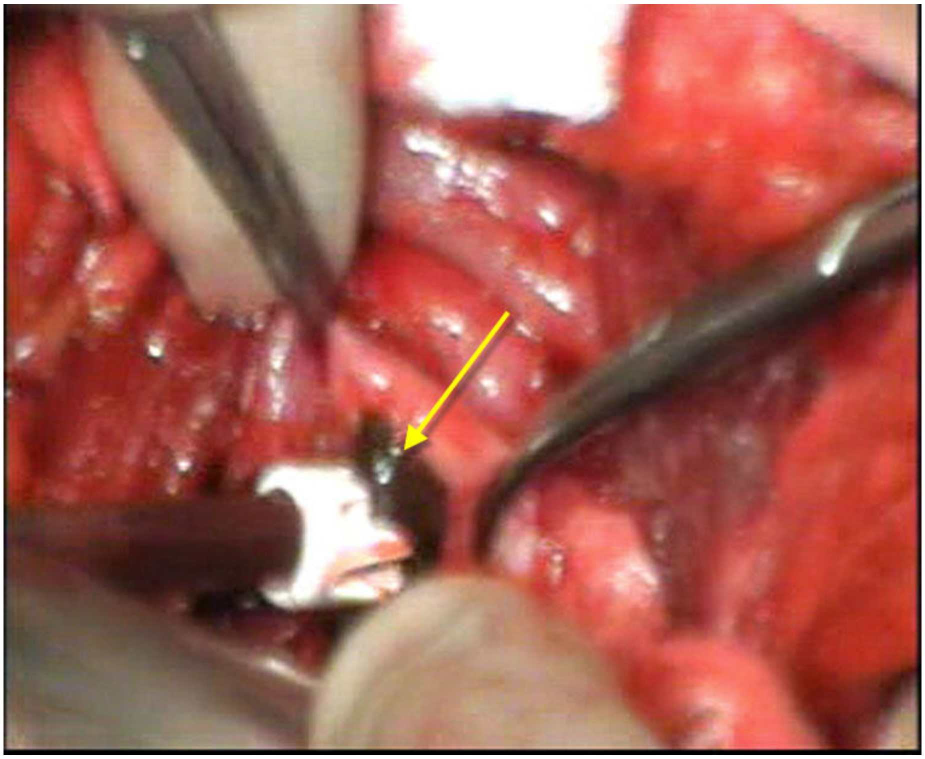

**FIGURE 5.** Chocolate cyst; thick brown fluid content.

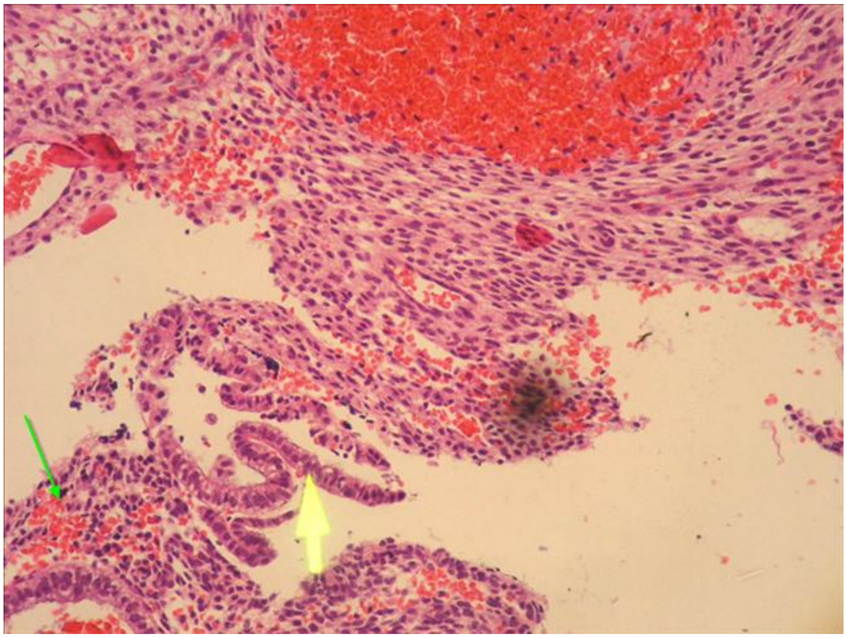

**FIGURE 6**. Microphotograph 40 × 10, showing endometrial glands, hemosiderin-laden macrophage, and areas of hemorrhage. Features of endometriosis.

ureteric involvement in endometriosis should be suspected in patients who complain of suprapubic or flank pain, frequency, urgency, dysuria, or hematuria. Ureteric involvement is uncommon, most of the time silent, and usually diagnosed late, often resulting in loss of the renal unit. In spite of the low percentage of patients with urinary tract involvement, it is reported that nearly 30% of these patients require a nephrectomy for a hydronephrotic or nonfunctioning kidney[2]. A familial incidence of endometriosis by a polygenic/multifactorial mechanism has been documented. A female patient with an affected first-degree relative has an approximately tenfold-increased risk for developing the disease[3]. The diagnosis of endometriosis is based on history, physical examination, and confirmed by laparoscopy and biopsy. On bimanual vaginal and rectal examination, tender nodules can be felt in the posterior vaginal fornix or along the uterosacral ligaments. Endometrial lesions have a blue or black powder burn appearance, but they can also be red, black, white, and nonpigmented. USG, CT, and MRI can be helpful adjuncts to the diagnosis and in ruling out the presence of other pelvic pathology, ureteric and bowel involvement. The Müllerian antigen CA-125 (>35 U/ml) is found to be elevated in 30–50% of stage-III or -IV disease, but has no significant value as a screening tool. However, CA-125 is a valuable adjuvant when following patients who had an initially elevated CA-125[4]. In our case, the values decreased from 86–41 U/ml after excision of the lesions. Treatment of endometriosis depends on the presentation, age, future fertility need of the patient, and the severity of the disease. Options for treatment can be categorized as definitive or palliative. Definitive therapy includes excision of all significant endometriomas and hysterectomy with bilateral oophorectomy. Patients with severe or moderate disease may benefit most from surgical therapy. The goal of medical therapy is to stop ectopic endometrial tissue growth via hormonal suppression. Medical management includes the use of combined estrogen-progesterone regimens, danazol, and gonadotropin-releasing hormone agonists (GnRHa) with or without add-back therapy. GnRHa cause LH and FSH levels to decrease, causing ovarian steroidogenesis to reversibly fall to menopausal level[5]. The successful resolution of a patient's symptoms, pain, or infertility is evident within 2–3 months of treatment, but this does not denote improvement in the urologic manifestation of the disease.

Radiographic evaluation is essential for the proper diagnosis and treatment of UE. The level and degree of obstruction and, most importantly, the relative function of the kidneys must be established preoperatively. Appearance of UE in IVU is nonspecific, resembling a stricture or tumor and typically appearing as a short, tight stricture in the pelvic ureter 2–5 cm above the ureteral orifice[6]. USG, CT, and MRI help in identifying the extent of disease. MRI is the imaging modality of choice for pelvic and UE. It has been reported to identify lesions that are 4 mm and larger. UE appears as a hypointense nodule on T2-weighted images and hyperintense foci on T1-weighted images. It helps in identifying extrinsic and intrinsic UE[7].

Treatment of UE, in general, should be surgical. Medical therapy rarely relieves the periureteral scarring and fibrosis. Nishihara et al., in their study, treated 4/8 cases of UE with GnRHa and found that the therapy was effective in only 2 patients. There was recurrence of hydronephrosis in them also, finally needing surgical management[8]. Present literature does not support the use of hormonal therapy alone for UE. Hormonal therapy fails to reverse or to stop the progression of ureteral stenosis[9]. Current therapeutic recommendations for women with significant UE who do not desire further pregnancies include resection of the endometriosis, bilateral oophorectomy, with or without hysterectomy. Ureteric obstruction has to be relieved by ureterolysis or resection of the involved segment followed by ureteroneocystostomy or uretero-ureterostomy and postoperative hormonal therapy. Some authors advocate only ureteric dilatation or ureterolysis for low-stage disease. A recent study showed that laparoscopic ureterolysis and removal of associated adenomyotic lesions was sufficient therapy in 16/18 cases of UE and only two required resection of the ureteral stenotic segment[10]. Psoas Hitch, a Boari flap, transuretero-ureterostomy, or even an ileal interposition may be used when necessary. If the ipsilateral kidney is severely impaired, then nephrectomy is the procedure of choice. In those who desire future fertility, conservative surgery and/or hormonal therapy is often recommended. Careful follow-up will be necessary in this patient population to ensure that obstruction does not recur.

We did a retrospective review of all the operated cases for the last 4.5 years at Abha Maternity Hospital, Assir. A total of 8684 operative procedures (obstetric and gynecology) were carried out during this time. This was the only case of UE apart from two other cases of extensive pelvic endometriosis wherein subtotal hysterectomy with bilateral oophorectomy was done. There was no urinary tract involvement in either of them. Similarly a retrospective review of the urological operative registry confirmed this to the only case of UE registered for the last 5 years at Assir Central Hospital.

## CONCLUSION

The unique features in our case are noteworthy: primary presentation of pelvic endometriosis in an unmarried female with a first-degree relative of endometriosis, complaining of vague loin pain to the urologist, with only an incidental finding of obstructed kidney on IVU and absence of menstrual irregularities. Also notable in this case is the early diagnosis of UE, initial primary hormonal monotherapy, and its failure to relieve ureteric obstruction in spite of 6 months continuous treatment. Initial attempts at dilatation of the ureteric stricture were fruitless and only surgical excision of the stricture helped to salvage the kidney.
